# Supplementary material for: Melittin-induced alterations in morphology and deformability of human red blood cells using quantitative phase imaging techniques
Source: Sci Rep. 2017 Aug 24;7:9306. doi: 10.1038/s41598-017-08675-7 (PMC5571175; doi:10.1038/s41598-017-08675-7)
Supplement: Supplementary file 1 — Supplementary Information [file 41598_2017_8675_MOESM1_ESM.pdf]

Melittin-induced alterations in morphology and deformability of human red blood cells using quantitative phase imaging techniques

Joonseok Hur[1,4], Kyoohyun Kim[1,2], SangYun Lee[1,2], HyunJoo Park[1], and YongKeun Park[1,2,3*]

[1]Department of Physics, Korea Advanced Institute of Science and Technology, Daejeon 34141, Republic of Korea.
[2]KAIST Institute Health Science and Technology, Daejeon 34141, South Korea
[3]Tomocube Inc., Daejeon 34051, Republic of Korea
[4]Current Affiliation: Department of Physics, Massachusetts Institute of Technology, Cambridge 02139, United States

[*]Corresponding Authors: yk.park@kaist.ac.kr (Y.P.)

Supplementary Information

Correlation between dimple and cell volume of RBC

The relation between change in cell volume of RBC and existence of dimple was investigated through analysis of curvature. The local curvature maps of RBCs obtained from 2-D phase images were analyzed of RBCs to find whether RBCs had dimples or not (Fig. S1). For a discocyte, mean curvature, defined by $H(x,y) = [\kappa_{max} + \kappa_{min}](x,y)/2$ where κ_{max} and κ_{min} are maximal and minimal curvatures, had positive values at the dimple region, while spherocyte had negative values of mean curvature (Figs. S1A-B). By taking an average of mean curvature in the circular area whose center is the center of mass of phase image of each RBC and radius is 30% of the radius of RBC, the existence of dimple in RBCs was investigate at various concentrations (Fig. S1C). The mean values of averaged mean curvature were positive at 0 nM to 150 nM, but the values became negative from 200 nM, which means the loss of dimple in RBCs. The mean values were 0.200 ± 0.100, 0.038 ± 0.165, 0.124 ± 0.136, 0.049 ± 0.184, -0.173 ± 0.235, -0.175 ± 0.231, and -0.397 ± 0.208 μm^{-1} at each melittin concentration. Because of the melittin concentration at the loss of dimple, between 150 nM and 200 nM, coincided with the concentration of the decrease of cell volume (Fig. 2A), we suggest a correlation between the loss of dimple as a change of global structure and the decrease of cell volume.

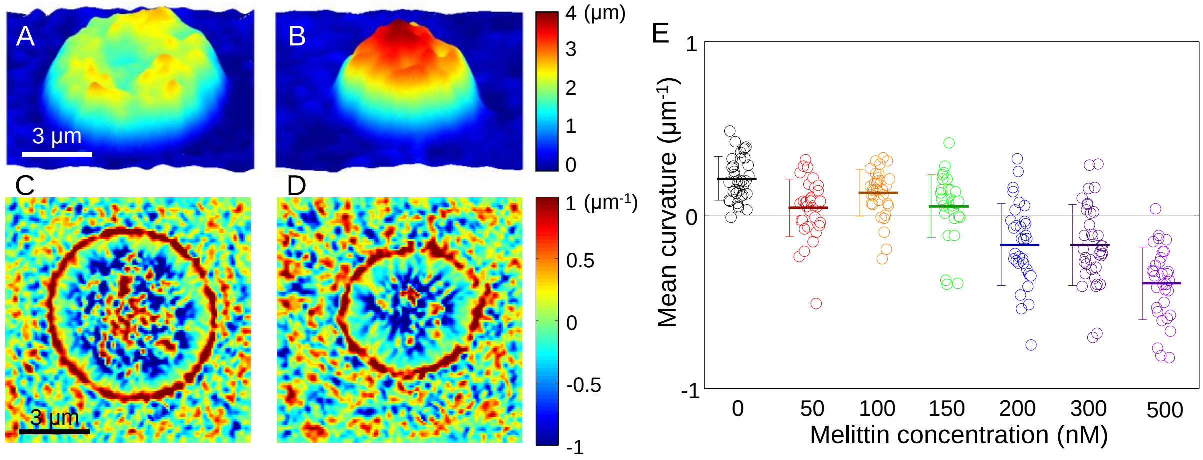

Fig. S1 (A-B) 2-D topographical images of *A* discocyte at 0 nM and *B* spherocyte at 500 nM. (C-D) Corresponding mean curvature maps of A-B, respectively. (E) Averaged values of mean curvature in dimple sites of individual RBCs at various melittin concentrations of 0, 50, 100, 150, 200, 300, and 500 nM.

Melittin-induced Hb diffusion in RBCs

Observation of Hb diffusion at high melittin concentrations was repeated at 5 μM and 10 μM of melittin concentrations (Fig. S2). Characteristic times of exponential decay were fitted from the measured dry masses over time during Hb leakage, and total areas of pores and numbers of pores were estimated (Table S1). As melittin concentration increased, the leakage of Hb became faster; characteristic time decreased, and total area and the number of pores increased as expected.

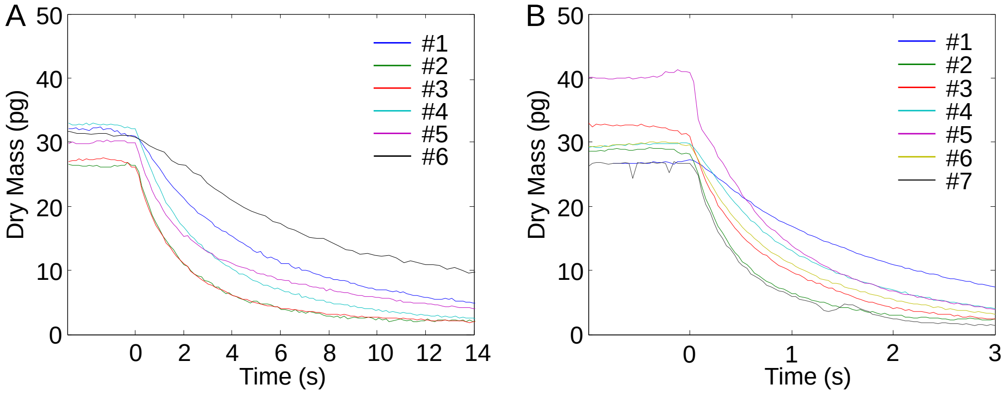

Fig. S2 Changes in dry masses of RBCs over time during Hb leakage at melittin concentrations of 5 μM (A) and 10 μM (B). The first data in A (blue line) is the same with the data in Fig. 7E (red line).

	τ (s)	A_{pore} (nm^2)	N_{pore}
5 μM	4.85 ± 1.58	2061 ± 632	105 ± 32
10 μM	1.19 ± 0.49	8675 ± 2890	442 ± 147

Table S1 Mean values and standard deviations of characteristic time of exponential decay (τ), total area (A_{pore}), and a number of pores (N_{pore}) at melittin concentrations of 5 μM and 10 μM.
